# Atypical Presentation of Infective Endocarditis With Native Aortic Valve Involvement Secondary to Staphylococcus aureus Bacteremia in the Setting of Non-Hodgkin Lymphoma

**DOI:** 10.7759/cureus.29012

**Published:** 2022-09-10

**Authors:** Omar Rafa, Eric J Basile, Arroj Ali, Prutha D Patel, Leonard Palatnic

**Affiliations:** 1 Internal Medicine, University at Buffalo, Buffalo, USA; 2 Internal Medicine, University of Florida College of Medicine, Gainesville, USA; 3 Cardiology, University at Buffalo, Buffalo, USA

**Keywords:** valvular endocarditis, aortic valve disease, staphylococcus aureus bacteremia, staphylococcus aureus endocarditis, infective endocarditis

## Abstract

Infective endocarditis (IE) is a condition that can involve endocardial tissue and possibly lead to valvular disease. Not only is it important to recognize the clinical presentation difference between acute and subacute IE, but physicians should understand that underlying risk factors, such as immunosuppression secondary to non-Hodgkin lymphoma (NHL), can alter an acute presentation into that of a subacute one. We report an NHL patient, who presented with subacute clinical symptoms of IE, but had a clinical test workup that showed evidence of acute IE rather than subacute.

## Introduction

Infective endocarditis (IE) is an infection that involves the innermost layer of the myocardium, the endocardium. The number of hospitalizations in the United States reported annually due to this condition is 12.7 per 100,000 [1]. The clinical presentations from IE are broken down into two general categories: acute and subacute. The former presents with more pronounced clinical details such as sustained fever and septic bacteremia over the course of days to weeks [2]. The latter involves a larger time span that can even reach months before a patient will tend to show symptoms such as weight loss, fatigue, and shortness of breath [1,2]. These presentations are predetermined by various factors. The severity increases with certain risk factors and microbes.

Risk factors for IE include cutaneous lesions, dental procedures, poor dental hygiene, structural endocardial and valvular defects, history of prior IE, patients who are immunosuppressed, injection drug users, and an age greater than 60 [3]. The most common entry etiologies, in descending order, are cutaneous (40%), oral (29%), and GI (23%) [4]. In terms of microbiology etiologies, the clinical presentation of acute presentations most often tends to be associated with Staphylococcus aureus while those of subacute is associated with Streptococcus viridans [2].

Non-Hodgkin lymphoma (NHL) is another risk factor that can potentially play a role in IE presentation alteration. NHL is a condition that involves inappropriate proliferation of immature and mature B and T cells, particularly in the lymphoid tissues [5]. Specifically, NHL leads to immunosuppression due to two reasons: the treatment and the condition itself. The former is usually chemotherapy, which directly targets the proliferation of immuno-protective cells. The latter reason can be explained by NHL’s involvement in the proliferation of immature cells, which can lead to vulnerability to infections [5]. No matter the case, both factors can lead to myelosuppression, sometimes neutropenic fevers, and can alter presentations of IE making it hard to clinically determine if it is an acute or subacute case on initial planning for medical management.

## Case presentation

On day 1 of the encounter, a 77-year-old Caucasian male presented to the emergency room (ER) with a chief complaint of chest pain, fatigue, and joint pains for the past couple of months. The patient reported that he was fixing his garage door when the chest pain started that day. The pain was described as sharp and transitioned to pressure-like. He pointed to the right side of his chest, graded the pain 6/10, and stated that it occurred on and off. He states that lying down and deep inspirations made the pain worse while taking a resting seat position alleviated it. He also stated he has had pain in his right calf for the last eight months, weight loss of 20 pounds for the past two months, and cold sweats with diffuse joint pains for the last six months. He also recalled a failed tooth extraction that led to the refilling of molar 30 two years ago. He denied nausea, chills, muscle aches, coughs, past hospitalizations, tobacco, and illicit drug use. The patient has a past medical history of unprovoked deep vein thrombosis and pulmonary embolism five months prior to the visit and non-Hodgkin lymphoma (NHL). The patient’s only home medication included 150 milligrams (mg) of dabigatran twice a day.

In the ER, the patient’s vital signs were stable except for a one-time spiked fever of 100.4 degrees Fahrenheit (F). A comprehensive physical exam was benign. An oral examination showed no signs of inflammation nor active infection. An electrocardiogram (ECG) showed normal sinus rhythm with regular intervals and no signs of ischemic changes. Comprehensive metabolic panel, urinary analysis, and double troponin levels (eight hours apart) were negative. A comprehensive blood count was remarkable for pancytopenia without bandemia. A CT angiography (CTA) study was performed and ruled out pulmonary embolism. A COVID-19/SARS rapid-antigen test was performed and came back negative. A 2-view anterior-posterior chest X-ray (CXR) was performed and was unremarkable. A transthoracic echocardiogram (TTE) was performed and showed a left ventricular ejection fraction (LVEF) of greater than 55% with moderate dilatation of the right ventricle (RV) and inferior vena cava (IVC). One set of blood culture (12 hours apart) was drawn, and the patient was stared on ceftriaxone 1 gram (g) intravenously (IV) once a day. The patient was administered metoprolol succinate 12.5 mg, atorvastatin 80 mg, aspirin 81 mg, per oral (po) once daily. He was given sublingual nitroglycerin 0.4 mg as needed and a 5,000-heparin bolus IV with additional acute coronary syndrome (ACS) heparin dosing. The patient was admitted for further evaluation and monitoring of possible ACS.

**Figure 1:**
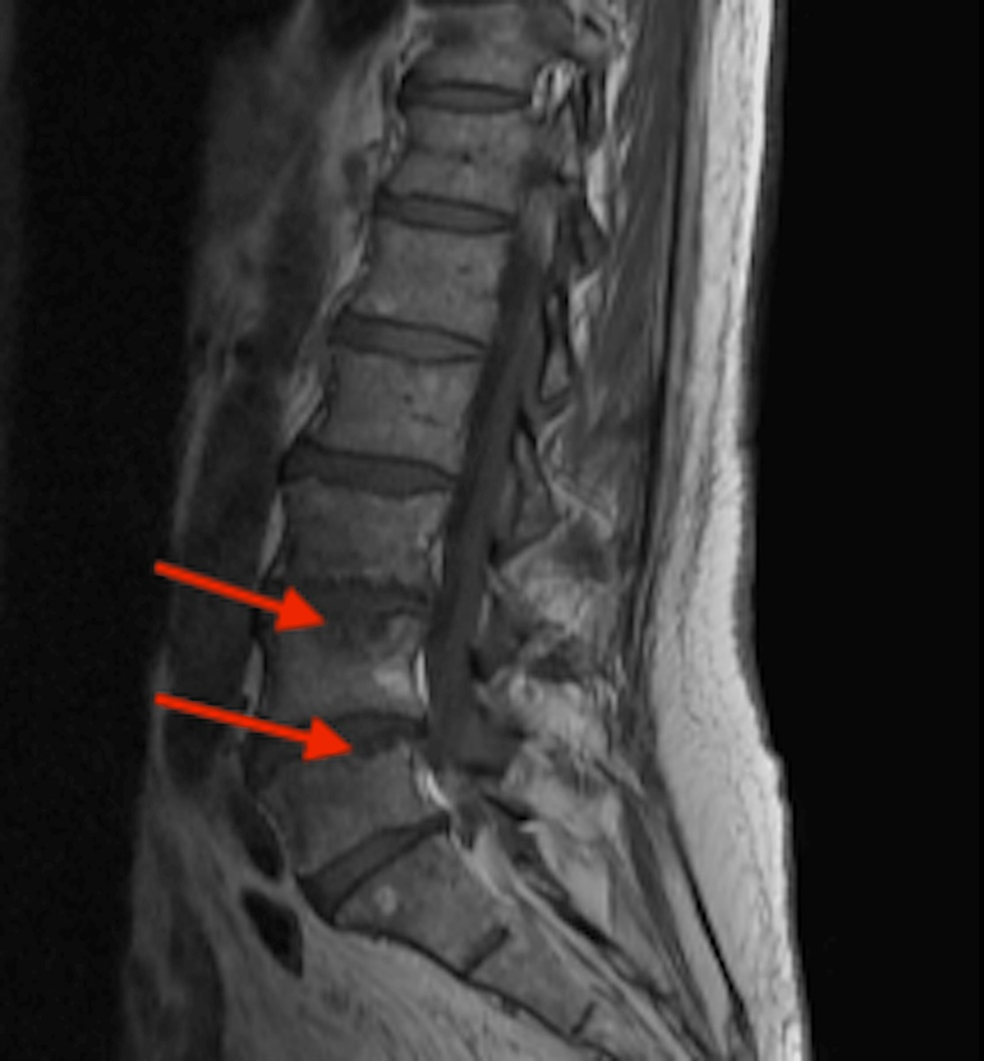
Magnetic resonance imaging depicts discitis L4-5 vertebrae (red arrows).

**Figure 2:**
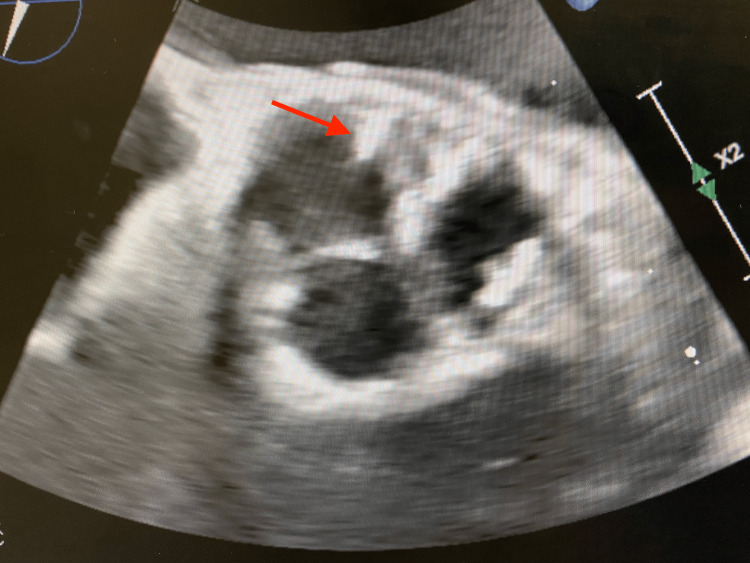
Transesophageal echocardiogram depicts vegetation on the aortic valve (red arrow).

On day 2, a lower extremity ultrasound was obtained and showed chronic deep vein thrombi. A second set of blood cultures were drawn. A third troponin test was negative. On day 3, a nuclear stress test was performed and came back negative for possible ischemic pathology. The patient complained of diffuse joint pains and was administered ibuprofen 600 mg orally, which helped alleviate the pain. He spiked a fever of 101 degrees F. All medications were discontinued except for antibiotics. His home dabigatran was restarted. On day 4, the first set of blood cultures were positive for Staphylococcus aureus (SA). The patient’s antibiotic regimen was changed to vancomycin 1.2 g IV twice a day. Magnetic resonance imaging (MRI) of the spine showed findings consistent with discitis on the L4-5 vertebrae depicted in Figure 1. On the fifth day, the patient received a transesophageal echocardiogram that showed aortic valvular vegetation where the right and left semilunar cusps meet, measuring less than 0.5 cm. This can be seen in Figure 2. Oral X-rays were obtained and ruled out acute infectious processes.

On the sixth day, the second set of blood cultures were negative. The patient’s vitals were stable. The decision was made to discharge the patient on cefazolin 2mg IV three times a day after the placement of a peripherally inserted central catheter (PICC) line. The patient’s comprehensive blood count remained in pancytopenia without bandemia throughout the remainder of the visit. The patient was notified to follow up with his outpatient oncologist and cardiologist.

## Discussion

The presentation of acute IE in this patient was atypical because of three main issues: aortic valve involvement in the setting of SA bacteremia, low-suspicion presentation for subacute IE, and lack of microbe-entry etiology. One study found the risk of endocarditis in patients with SA bacteremia to be as low as 5% [6]. As low as the probability of incurring IE in the setting of SA bacteremia may be, it is an important clinical differential, which needs to be maintained, when a patient’s blood culture comes back positive SA. This ties back to our case because his cardiac exam was benign throughout his hospital stay. Having a benign physical exam cannot rule out IE, especially in the face of positive SA bacteremia.

In terms of the presentation, there is also a second issue to consider here: the presentation was that of subacute IE in this patient: months of fatigue, weight loss, and joint pains. There was low suspicion for IE given the benign heart exam and stable vitals except for one spiked low-grade fever. One meta-analysis from 2014 found a 90-day mortality rate in roughly 30% of the patients with SA bacteremia [7]. This, essentially, validates the notion that SA bacteremia with IE involvement should be kept as a possibility despite mild symptom presentation, considering the remaining, estimated, 70% of SA bacteremia patients are not included in the 90-day mortality rate and will likely not present in critical condition. A 10-year Denmark study analyzed the clinical features of SA endocarditis that included 260 total patients, excluding drug addicts [8]. The study found that endocarditis was not clinically suspected on initial presentation in 83 of them. Furthermore, it found that the mitral valve was more commonly affected than the aortic valve [8]. This further adds merit to our case given that he had aortic valve vegetations with mild and unspecific symptoms (fatigue, weight loss, and cold sweats).

In terms of initial source of microbe-entry, skin sources are the most common method. They almost account for half of the total etiologies of IE [4]. However, this patient was thoroughly inspected and questioned about skin lesions, catheters, indwelling devices, past surgeries, and any other skin breaks. Nothing alarming in terms of cutaneous involvement was suspected. Despite having a history of dental procedure, we do not believe there is an association due to the procedure being done two years ago and benign oral and X-ray exam. Another reason is that most incubation period for IE post-procedure for invasive dental procedures is between 7-14 days [9]. Due to the lack of clear etiological evidence, the original source of blood entry is unknown.

Regardless, one fact remains particularly important here: recognizing IE in this case was difficult because this patient was immunosuppressed. The last positron emission tomography (PET)/computed tomography (CT) scan, which was performed five months prior to the visit, revealed hypermetabolic bilateral hilar and mediastinal lymphadenopathy and a small focus of hypermetabolic activity within the left internal mammary lymph node region that was not clearly present on the previous study, which was done seven years ago. This information, coupled with the fact that he had pancytopenia throughout the hospital stay, leads us to suspect immunosuppression can mask the symptoms and lab values of a patient with an underlying bacteremia. Interestingly, one study found that, among bacteremia patients, nearly 52% had a normal white blood cell (WBC) count while 80% displayed bandemia, which is basically greater than 5% of WBC w/differential [10]. Although this suggests bandemia might be a better indicator to access for bacteremia, our patient did not have an elevated WBC count nor bandemia while still having SA bacteremia with IE. This piece of information from our case confirms the need for a higher-than-normal suspicion for IE in immunosuppressed patients with bacteremia.

This patient benefited from medical management over surgery for a number of reasons. The primary indications for surgery in IE patients are as follows: abscess accumulation, pseudo aneurysms, mechanical valvular breakdown, decompensated heart failure leading to systemic vascular congestion, and a vegetation with a diameter of 0.5 cm or higher [4]. The size of the valve in our patient was no more than 0.5 cm and he did not possess any other criteria for surgical management.

## Conclusions

The presentation of acute infective endocarditis (IE) can be altered if the patient has a history of immunosuppression, specifically non-Hodgkin lymphoma. Timely blood cultures and empiric coverage can have a large impact on patient care outcomes. This case emphasizes and reaffirms that symptom severity does not necessarily correlate with disease severity in immunosuppressed patients. Patients with Staphylococcus aureus bacteremia may benefit from a transesophageal echocardiogram regardless of symptom presentation.
